# Common mental disorders in mothers of children attending out-patient malnutrition clinics in rural North-western Nigeria: a cross-sectional study

**DOI:** 10.1186/s12889-021-10227-8

**Published:** 2021-01-21

**Authors:** Aminu T. Abdullahi, Zubaida L. Farouk, Abdulazeez Imam

**Affiliations:** 1 Department of Psychiatry, Bayero University Kano, Kano, Nigeria; 2 Department of Psychiatry, Aminu Kano Teaching Hospital, PMB 3452, Kano, Nigeria; 3 Center for Infectious Diseases Research, Bayero University Kano, Kano, Nigeria; 4 Department of Paediatrics, Aminu Kano Teaching Hospital, PMB 3452, Kano, Nigeria; 5 Department of Vaccines and Immunity, Medical Research Council Unit The Gambia at the London School of Hygiene and Tropical Medicine, P.O. Box 273, Atlantic Boulevard, Fajara, Gambia

**Keywords:** Nutritional status, Childhood malnutrition, Mental health, Nigeria, Developing countries

## Abstract

**Background:**

Children with uncomplicated severe acute malnutrition are managed routinely within out-patient malnutrition treatment programs. These programs do not offer maternal mental health support services, despite maternal mental health playing a significant role in the nutritional status of children. Additionally, the burden of maternal Common Mental Disorders (CMDs) is poorly described among mothers of children attending these programs.

This study thus determined the burden and risk factors for maternal CMDs among children attending out-patient malnutrition clinics in rural North-western Nigeria.

**Methods:**

We conducted a cross-sectional study among 204 mothers of children with severe acute malnutrition who attending eight out-patient malnutrition clinics in Jigawa, North-western Nigeria. We used the World Health Organization Self-Reporting Questionnaire-20 (WHO SRQ-20) screening tool, a recognised and validated proxy measure for CMDs to identify mothers with CMDs. The prevalence of maternal CMDs was determined by identifying the proportion of mothers with SRQ scores of ≥8. Risk factors for CMD were determined using multivariable logistic regression.

**Results:**

Maternal CMD prevalence in children attending these facilities was high at 40.7%. Non-receipt of oral polio vaccine (OPV) (AOR 6.23, 95%CI 1.85 to 20.92) increased the odds for CMD. While spousal age above 40 (AOR 0.95, 95%CI 0.90 to 0.99) and long years spent married (AOR 0.92, 95%CI 0.85 to 0.98) decreased the odds for CMD.

**Conclusions:**

Our findings indicate maternal CMD burden is high in out-patient malnutrition clinics in North-western Nigeria. Maternal mental health services would need to be integrated into the community management of acute malnutrition programs to provide more holistic care, and possibly improve long-term outcomes after discharge from these programs.

## Background

Childhood acute malnutrition is an important underlying cause of under-five mortality in low and middle-income countries (LIMCs), where its severe form, severe acute malnutrition (SAM) is associated with 174,000 annual deaths [1]. In these settings, endemic poverty, and health inequalities have been identified as some background causes of a high acute malnutrition burden [2].

Maternal factors are also important determinants for childhood acute malnutrition. Physical and social factors such as maternal stature, socioeconomic status, family structure, and intelligence have been implicated in its aetiology and are extensively studied in LMIC settings [2–5]. Maternal psychological factors, although closely related to childhood malnutrition, have received less attention in these settings, and this is typified by only 6% of published global mental research coming from LMICs [6]. This is also despite a disproportionately high burden of both acute malnutrition and Common Mental Disorders (CMDs) such as anxiety and depression in these settings [7].

The CMDs share similar risk factors with acute malnutrition; for example, poverty and social deprivation [8, 9]. There is probably a bi-directional relationship between both entities. Mothers of malnourished children are known to show increased risks for CMDs [10], while population-based studies demonstrate increased risk for acute malnutrition in infants of mothers with CMDs [11–14]. With the existing evidence of an association between childhood acute malnutrition and maternal CMDs from population studies [15], it is important to identify whether maternal CMD is highly prevalent in acute malnutrition treatment programs. The presence of a high burden can potentially negatively affect the successful outcomes of these programs.

Children with SAM are treated within the community management of acute malnutrition (CMAM) programs in many LMICs [16]. These provide decentralised out-patient treatment for acute malnutrition. In Nigeria, 6 out of 11 state CMAM programs are located in the North-western region because of its high acute malnutrition burden [17]. The success of these treatment programs relies on the provision of holistic care focusing on both proximate and distal factors such as maternal mental state, that are associated with acute malnutrition. These out-patient treatment programs do not have any maternal mental health programs, and to the best of our knowledge, the burden of maternal CMDs in these programs has not been systematically evaluated. Only a few studies have previously investigated maternal CMDs within acute malnutrition treatment programs that were in-patient rehabilitation units [18–20], and these contexts might differ from those of out-patient malnutrition centres.

**Fig. 1:**
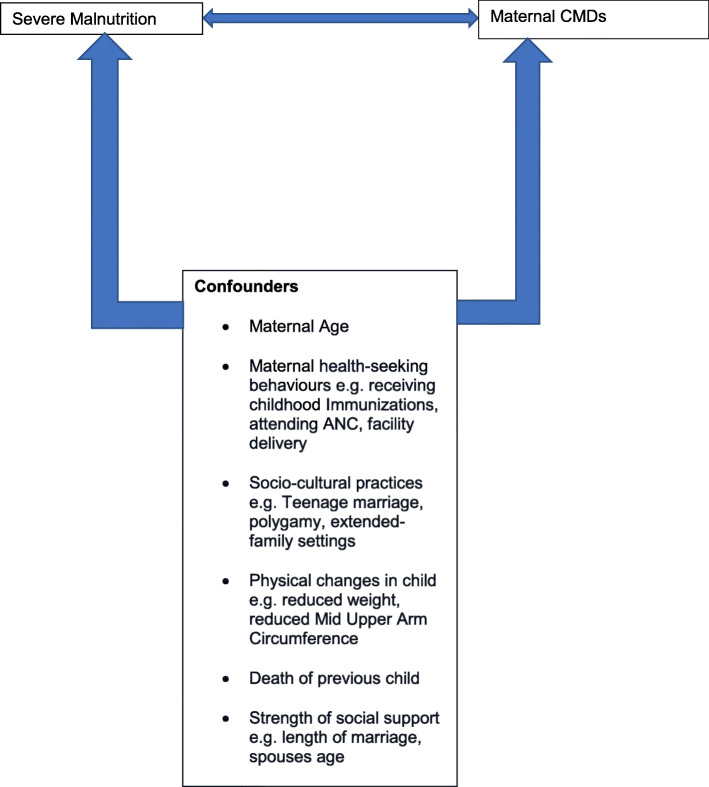
A conceptual framework for severe malnutrition and Maternal CMDs

Our study thus determined the burden and risk factors for CMDs among mothers of children with SAM who were enrolled in out-patient acute malnutrition treatment programs in rural Northern-western Nigeria. In addition to previously described risk factors for maternal CMD in acutely malnourished children, we investigated possible associations with locally prevalent socio-cultural factors (using the conceptual framework in Fig. 1), such as a history of teenage marriage, living in polygamous households, or extended family households. This is because factors affecting mental health can vary significantly across geography and cultural contexts [21].

We hypothesize that the burden of maternal CMDs in local acute out-patient malnutrition treatment programs is high in North-western Nigeria, and might be associated with common local socio-cultural practices (Fig. 1). We hope our findings provide evidence that highlights the need for the provision of integrated services within CMAM that take into cognisance maternal mental well-being.

## Methods

### Study design and setting

We performed a cross-sectional study using the World Health Organization Self-Reporting Questionnaire-20 (WHO SRQ-20) scores as a proxy measure for maternal CMDs among children with SAM [22]. These were under-five children attending CMAM out-patient malnutrition clinics in Jigawa State, North-western Nigeria. We also identified risk factors for an SRQ score ≥ 8, which we identified as having a CMD [15, 18].

The Jigawa state CMAM program is one of 11 CMAM programs run in northern Nigeria and is a collaboration between the Nigerian Federal Ministry of Health and the United Nations International Children’s Emergency Fund (UNICEF). Its objective is to improve local coverage for the treatment and control of severe acute malnutrition in Nigeria. Jigawa state has a population of about 5.5 million people, and the state has 16 CMAM malnutrition clinics across 5 of its 16 Local Government Areas (LGAs). The predominant language of communication in this setting is the Hausa language.

We conducted our study in eight of the 16 CMAM clinics, and these were located in Jahun Babura and Maigatari LGAs in rural Jigawa. Our study clinics were Aujara, Jarmai, Kadowawa, Tashar Dan kyambo, Takwasa, Kanya, Garu, and Jahun. At the time of conducting our study, average clinic attendance was between 20 and 30 newly enrolled children with SAM per week.

### Study population

We enrolled mother and child pair attending CMAM program enrolment visits. We defined SAM using mid-upper arm circumference (MUAC) < 11.5 cm, based on World Health Organization (WHO) recommendations [23].

### Sample size determination

We calculated the sample size for proportions using the Open Epi statistical software [24]. We set our confidence level at 90%, precision at 5% and determined the proportion of mothers with common mental disorders as 23.3% based on a previously published study [19], determining a minimum sample size of 194. We added 5% to account for missing data deriving our sample size of 204.

### Sample selection and technique

We used a sampling ratio of 50% of our sampling frame (16 CMAM clinics) to arrive at the largest possible sample (8 clinics) for a chosen error margin of 0.05 [25]. We chose these 8 CMAM clinics using convenience sampling. Consecutive mother and child pair who gave consent for the study were enrolled in these clinics over 8 months from January 2014 to August 2014.

### Data collection

Trained research assistants collected study data, measured anthropometric indices of the children (MUAC and weight), and administered the WHO SRQ-20 to mothers. These research assistants were five community health extension workers assisted by 15 students of the School of Health Technology Jahun and they were independent of the local CMAM program. Before our study commencement, they were trained for 2 weeks on the use of the WHO SRQ-20 tool, administration of our questionnaire, and measurement of anthropometry. The weight of enrolled children was measured to the nearest 0.1 kg using a Mother-and-child scale (Salter®), while mid-upper arm circumference was measured using a UNICEF MUAC tape.

### Exposure variables

We collected data on the following variables:

- Child variables:
  Child’s age, gender, number of siblings, admission weight, MUAC, skin change and immunisation history (BCG, OPV and DPT vaccine administration).
- Maternal variables:
  Maternal age, employment, spouses age, years of marriage, antenatal care attendance, delivery place, death of a previous child,
- Family variables:
  Family setting and structure.

### Outcome variable

The SRQ-20 was developed by the World Health Organization (WHO) as a screening tool for assessing maternal mental health [22]. It is a recognised proxy measure for CMDs and has been validated in multiple developing country settings [26, 27]. It is a scale-based score consisting of 20 yes or no questions which ask about multiple symptoms related to experiences of depression, anxiety, somatic, and panic symptoms which have occurred within the preceding 30 days. When a symptom is present within the specified period, the individual is given a score of 1, while 0 represents the absence of symptoms within the same period. The sum of all the scores represents the overall SRQ-20 score and higher scores represent poorer maternal mental health. We considered a cut-off maternal SRQ score of ≥8 to define maternal CMD which was based on previously published work with similar analysis to ours [10, 15, 18]. We translated the WHO-SRQ-20 tool to the local dialect (Hausa) and back-translated to English to ensure accuracy. Following this, the Hausa version was used to access our outcome (maternal CMDs).

### Ethical considerations

Ethical approval for our study was obtained from the Institutional Review Board of Aminu Kano Teaching Hospital and informed consent was obtained from participants before study enrolment.

### Data analysis

We categorised our outcome data, maternal SRQ-20 scores into two groups: a score ≥ 8 which signified the presence of maternal CMD and we interpreted a score < 8 as the absence of CMD. We provided participant summary statistics using means and standard deviation for normally distributed data and median with interquartile range for non-normally distributed data. We compared maternal and child characteristics between those with high WHO SRQ scores (≥ 8) and those with low scores (< 8) using chi-square to compare proportions and the Man-Whitney U test to compare medians. Statistical significance was set at *p* < 0.05.

For multivariable logistic regression analysis, we selected a cut-off of *p* < 0.20 to identify significant variables from univariable analysis to include in our multivariable model. All selected covariates were entered into bivariable and multivariable logistic regression models to determine crude and adjusted odds ratios respectively. Multivariable models contained all covariates with *p*-value < 0.20 and each covariate was adjusted for all other covariates within the model. All statistical analyses were performed using STATA version 13.1 (StataCorp. 2019. Stata Statistical Software: Release 16. College Station, TX: StataCorp LP).

## Results

We administered our study questionnaire to and completed WHO SRQ-20 scoring for 204 mothers of children with SAM. We initially approached 207 mothers attending the clinic for the first time, three of these children were orphans with grandmothers as caregivers and they were excluded from the study. Of our 204 participants, 121 (59.3%) had SRQ scores < 8 while 83 (40.7%) had scores ≥8. The median SRQ score ± Interquartile range was 3 ± 13.

### Child characteristics compared between mothers with SRQ scores < 8 and those with ≥8

Table 1 shows the characteristics of the children with SAM compared between both SRQ score groups. Children did not differ significantly across age, gender, or the number of siblings they had. Mothers with high SRQ scores (≥ 8), on average had infants with lower weights when compared to those with low SRQ scores (5.5 kg versus 6.0 kg, Table 1, *p* = 0.003). Both groups, however, had similar Mid-upper arm circumference measurements (Table 1, *p* = 0.70) and did not differ significantly in the proportion of children with skin changes (Table 1, *p* = 0.41).

**Table 1:** Children’s characteristics compared between mothers with high SRQ scores (≥ 8) and those with low scores (< 8)

Variable	Maternal SRQ score < 8 (%) *n* = 121	Maternal SRQ score ≥ 8 (%) *n* = 83	*P*-value	Total (%) *n* = 204
**Median Age (IQR)**	13 (13) months	12 (15) months	0.39	12 (13.5) months
**Gender**				
Male	53 (45.3)	40 (50.0)	0.52	93 (47.2%)
Female	64 (54.7)	40 (50.0)		104 (52.8%)
**Median number of siblings (IQR)**	4 (4)	5 (4)	0.61	5 (4)
**Mean admission weight (SD)**	6.0 (1.8) kg	5.5 (1.1) kg	0.003*	5.7 (1.2) kg
**Median MUAC (IQR)**	10.5 (1) cm	10.8 (1.2) cm	0.70	10.5 (1) cm
**Skin Changes**				
Yes	37 (30.6)	21 (25.3)	0.41	58 (28.4%)
No	84 (69.4)	62 (74.7)		146 (71.6%)
**Received BCG vaccine**				
Yes	61 (52.1)	41 (49.4)	0.70	102 (51.0)
No	56 (47.9)	42 (50.6)		98 (49.0)
**Received DPT vaccine**				
Yes	62 (53.0)	38 (45.8)	0.32	100 (50.0)
No	55 (47.0)	45 (54.2)		100 (50.0)
**Received OPV**				
Yes	111 (94.9)	52 (62.7)	< 0.001^*^	163 (81.5)
No	6 (5.1)	31 (37.4)		37 (18.5)

*BCG* Bacille Calmette Guerin, *DPT* Diphtheria Pertussis Tetanus, *IQR* Interquartile Range, *MUAC* Mid Upper Arm Circumference, *OPV* Oral Polio Vaccine

*Significant

Immunization uptake was similar across both groups, except for Oral Polio Vaccine (OPV), where uptake was significantly higher among mothers with low SRQ scores (94.9% versus 62.7%, *p* < 0.001, Table 1).

### Maternal characteristics compared between mothers with SRQ scores < 8 and those with ≥8

Table 2 compares maternal characteristics between mothers with SRQ scores of < 8 and those with scores ≥8. Median maternal age was significantly lower among mothers with a high SRQ when compared to those with low SRQ scores (*p* = 0.0003). The high SRQ group also had a significantly larger proportion of teenage mothers. The proportion of gainfully employed mothers did not significantly differ between groups.

**Table 2:** Maternal characteristics compared between mothers with high SRQ scores (≥ 8) and those with low scores (< 8)

Variable	Maternal SRQ score < 8 (%) *n* = 121	Maternal SRQ score ≥ 8 (%) *n* = 83	*P*-value	Total (%) *n* = 204
**Median maternal age (IQR)**	26 (9.5)	23 (10)	< 0.001*	25 (10)
**Age of mother (Teen or not)**				
< 18	3 (2.5)	13 (15.7)	0.001*	16 (7.8)
≥18	118 (97.5)	70 (84.3)		188 (92.2)
**Gainfully employed**				
Yes	24 (20.5)	15 (19.2)	0.83	39 (20.0)
No	93 (79.5)	63 (80.8)		156 (80.0)
**Median Years of marriage (IQR)**	15 (16)	10 (8)	< 0.001*	14 (7)
**Spouses Age**				
< 40	43 (35.5)	45 (54.2)	0.008*	88 (43.1)
≥ 40	78 (64.5)	38 (45.8)		116 (56.9)
**Family structure**				
Nuclear	47 (48.5)	48 (64.9)	0.032*	95 (55.6)
Extended	50 (51.6)	26 (35.1)		76 (44.4)
**Family setting**				
Monogamous	55 (45.8)	39 (47.6)	0.81	94 (46.5)
Polygamous	65 (54.2)	43 (52.4)		108 (53.5)
**Antenatal care attendance**				
Yes	59 (52.7)	50 (61.0)	0.25	109 (56.2)
No	53 (47.3)	32 (39.0)		85 (43.8)
**Delivery Place**				
Home	18 (16.8)	11 (13.4)	0.52	29 (15.3)
Hospital	89 (83.2)	71 (86.6)		160 (84.7)
**Death of a previous child**				
Yes	69 (57.5)	53 (63.9)	0.36	122 (60.1)
No	51 (42.5)	30 (36.1)		81 (39.9)

*IQR* Interquartile Range

*Significant

Median years of marriage were higher in the low SRQ score group (15 years) when compared to those with higher SRQ scores (10 years) and this difference was statistically significant (*p* < 0.001). Those with low SRQ scores also had a significantly higher proportion of spouses aged over 40 (*p* = 0.008) and a significantly larger proportion of them lived with extended family (*p* = 0.032). Family type, antenatal care attendance, delivery place of the index child, and history of previous child death was similar between groups.

### Risk factors for high SRQ scores

The child’s admission weight was not a significant risk factor for a high SRQ score (AOR 0.74, 95%CI 0.54 to 1.00, Table 3). Mothers of children who had not received the OPV vaccine had a 6-fold increased odds for having high SRQ scores (AOR 6.23, 95%CI 1.85 to 20.92, Table 3). While spousal age ≥ 40 was associated with a 5% decreased odds for having a high SRQ score (AOR 0.95, 95%CI 0.90 to 0.99, Table 3). Similarly, each added year of marriage was associated with an 8% decreased odds for having a high SRQ score (AOR 0.92, 95%CI 0.85 to 0.98, Table 3).

**Table 3:** Crude and adjusted odds ratios with corresponding 95% confidence intervals of risk factors for high maternal SRQ scores in the recruited cohort (*n* = 204)

Variable	Crude odds ratios and 95% confidence interval	Adjusted odds ratios and 95% confidence interval^b^
**Child’s admission weight**	0.73 (0.57 to 0.94)	0.74 (0.54 to 1.00)
**Received OPV vaccine**		
No	11.03 (4.33 to 28.07)	6.23 (1.85 to 20.92)^a^
Yes	1	1
**Spouses Age**		
≥ 40	0.95 (0.92 to 0.98)	0.95 (0.90 to 0.99)^a^
< 40	1	1
**Family structure**		
Extended	0.51 (0.27 to 0.95)	0.60 (0.27 to 1.30)
Nuclear	1	1
**Years of marriage**	0.88 (0.83 to 0.93)	0.92 (0.85 to 0.98)^a^
**Maternal age (years)**	0.94 (0.91 to 0.98)	1.02 (0.95 to 1.09)
**Maternal age (Teen or Not)**		
≥18	0.14 (0.04 to 0.50)	0.61 (0.10 to 3.79)
< 18	1	1

^a^Significant adjusted odds ratio

^b^Each adjusted odds ratio is adjusted for all other variables in the model

## Discussion

This paper describes a high burden of maternal CMD in acute malnutrition treatment centres in rural North-western Nigeria. It also corroborates the finding that maternal social support (measured as a longer duration of marriage and being married to an older aged spouse) decreases the odds for maternal CMDs. While non-receipt of oral polio vaccine (OPV) in the child increased the odds for maternal CMDs.

In this study, the maternal CMD burden among clinic attendees was high at 40.7%. This is similar to reports from Brazil, where 34.0% of mothers of children identified as malnourished during health provider visits had CMD [10]. Our findings, however, differ significantly from a Malawian study which demonstrated a very high maternal CMD prevalence of 71% among mothers of children with SAM [18]. While our study population was children attending out-patient malnutrition clinics, the former study was conducted among populations who were admitted to in-patient malnutrition stabilisation units. Hospitalisation in itself can presumably increase the risk for CMDs and might account for these observed differences. Other studies conducted in a similar context to ours have also described a higher prevalence of maternal mental disorders among malnourished children who were either in-patient or attended regular out-patient clinics [20, 28, 29]. A recent study in Sudan demonstrated mothers of admitted children with SAM had a greater prevalence of depression when compared to mothers of admitted well-nourished children [20]. A 2nd study conducted in Bostwana also found similar findings among mothers of children attending welfare clinics [28], while a Brazillian study described an association between stunting and CMDs [29]. As out-patient malnutrition treatment programs provide no maternal mental health support, our findings of a high maternal CMD burden in the studied out-patient malnutrition clinics suggest a need for possible integration of these services. This need is further strengthened by the existing evidence from population studies which suggest maternal CMDs having a significant role in poor childhood nutrition status [15]. The current focus of out-patient treatment programs is currently the child and how the mother can help the child sustain weight gain, and there have been recent calls for these programs to provide more integrative services [30, 31]. Maternal CMDs such as depression is characterized by a lack of energy, diminished concentration, loss of interest in the environment, and social withdrawal and understandably interferes with a mother’s ability to cater for her children and can affect the successes of these treatment programs [32]. A recent study in North-western Nigeria reported relapse rates to be as high as 24% in the immediate 6 months after discharge from treatment [30]. Although we did not investigate a role for maternal CMD, It is plausible maternal mental state might play an important role in acute malnutrition relapse, and this will need to be investigated by longitudinal studies. Practically, the first step needed to incorporate maternal mental health components into malnutrition treatment programs needs to be the detection of these mental health issues and the provision of therapy. Conceivably, the detection of CMDs by specialist mental health professionals is impractical, as many of these settings do not have sufficient mental health professionals. Tools like the WHO-SRQ 20 used in this study can easily be adapted and local CMAM support staff trained on this. Some identified key staff within these programs could also be trained to manage and support mothers identified as having CMDs. There is promising data from a cluster randomized trial in Zimbabwe, where researchers have shown the feasibility and positive impact of such task shifting to lay health workers [33]. In addition, the recent Covid-19 pandemic has highlighted the power of virtual platforms in provding both education and training support and it might be possible to harness this to support CMAM staff. Feasibility and practicality of the use of such platforms in this context would need to be investigated by future research.

Consistent evidence in the literature shows that social support significantly reduces the incidence of CMDs [34, 35], and this was demonstrated in our study. We found two proxy measures of social support, that is, years of marriage and spousal age were associated with reduced odds for maternal CMD. While each additional year of marriage, decreased the odds of maternal CMDs by 18%, having a spouse over the age of 40 years decreased the odds by 7%. Marriage represents a stable relationship, while spousal age over 40 might be representative of a more mature and stable partner, all of which are important characteristics of quality social support.

Also, we found that non-receipt of oral polio vaccine (OPV) in the child was associated with increased odds for maternal CMD. This finding was not observed for other routine childhood immunisations. Community OPV administration in northern Nigeria differs from the administration of most other vaccines. Regular vaccines are typically received only during immunisation visits, but OPV administration in addition to being received during such visits are given during mass door-to-door polio vaccination campaigns [36]. Studies have shown receipt or engagement of a mother with a health program is highly influenced by her mental functionality [7], as this finding might be explained by mothers with CMDs being less likely to engage in mass immunisation activities.

We did not find any association between CMD and our investigated socio-cultural factors such as polygamy, extended family settings, and teenage marriage. A recent study, however, described an association between polygamy and SAM in North-western Nigeria and hypothesized child-rearing and feeding practices in the local polygamous households as possible factors for this [17]. Our findings of no association for maternal CMDs and the studied socio-cultural factors might be related to these practices being common and perceived as the normal way of life in rural North-western Nigeria. This is manifest in our study where for example greater than half of the households were polygamous. In such settings, CMDs might typically not occur when an event is considered routine.

### Strengths and limitations

We did not make a definitive psychiatric diagnosis of CMDs among the interviewed mothers but relied on using the WHO SRQ-20 which is a proxy scale-based measure for CMDs. While the tool cannot replace conventional mental disorders, it is useful as a screening tool to identify probable cases of CMDs. One limitation of the tool however is that its validity and reliability can vary across multiple settings and languages and this requires adapting and validating it in different settings [37]. The WHO-SRQ 20 has however previously been validated in Nigeria and other similar developing country settings [26, 27, 38]. Also, we administered the tool in Hausa, the local dialect, following translation and back-translation to improve its accuracy and this would have aided participant understanding and limited response bias. A 2nd limitation of the tool is its non-specificity and its inability to separate individuals with depression from those with anxiety [15]. This limited our study inferences for the individual types of maternal mental disorders within out-patient malnutrition units and this would need to be answered by future research using more specific diagnostic criteria.

In addition, due to our cross-sectional design, we could not explore a role for maternal CMDs in weight attainment within these programs which was described in a previous longitudinal study [18]. We, however, expanded our investigation for potential local socio-cultural risk factors for maternal CMDs within these programs. Further longitudinal studies would be needed to determine what role maternal CMDs play in relapse rates within these programs. Also, our method of CMAM site selection was convenience sampling which is a non-probability method of sample selection with some risk of selection bias. Collecting data from eight out of the 16 malnutrition clinics in our setting would have limited any effects of intra-clinic variability on our data and allowed for a more community representative sample. We have also conducted this study within the community management of acute malnutrition (CMAM) program, which allows for more generalisable and improves on previous hospital-based studies [18, 39].

## Conclusion

Maternal CMD burden is high among children attending malnutrition clinics in rural Northern-western Nigeria. While social support factors reduced the odds for maternal CMDs, non-receipt of OPV vaccines increased the odds. We found no associations between common socio-cultural practices and maternal CMDs.

For malnutrition treatment programs to comprehensively tackle SAM and improve their success rates, these programs would need to provide greater holistic care to entrants that include providing support for maternal mental wellbeing.

## Data Availability

Data used in this study are available from the corresponding author upon reasonable request.

## Abbreviations

AOR: Adjusted odds ratio
BCG: Bacille Calmette Guerin
CMAM: Community management of acute malnutrition
CMDs: Common mental disorders
DPT: Diphtheria Pertussis Tetanus
IQR: Interquartile range
LGAs: Local Government Areas
LMICs: Low and middle-income countries
MUAC: Mid upper arm circumference
OPV: Oral polio vaccine
SAM: Severe acute malnutrition
WHO: World Health Organization
WHO SRQ-20: World Health Organization Self-Reporting Questionnaire-20
UNICEF: United Nations International Children’s Emergency Fund
